# Design, synthesis and biological evaluation of erlotinib-based IDO1 inhibitors

**DOI:** 10.3389/fphar.2022.940704

**Published:** 2022-08-10

**Authors:** Xi-xi Hou, Xiao-qing Gong, Long-fei Mao, Ge Sun, Jian-xue Yang

**Affiliations:** ^1^ The First Affiliated Hospital, and College of Clinical Medicine of Henan University of Science and Technology, Luoyang, China; ^2^ College of Chemistry and Chemical Engineering, Lanzhou University, Lanzhou, China; ^3^ The Third Affiliated Hospital of Guangzhou University of Chinese Medicine, Guangzhou, China; ^4^ Cancer Research Institute, School of Basic Medical Sciences, Southern Medical University, Guangzhou, China; ^5^ School of Nursing, Henan University of Science and Technology, Luoyang, China

**Keywords:** erlotinib, 1,2,3-triazole, IDO1, Attentive FP, antitumor

## Abstract

Erlotinib is a highly specific and reversible epidermal growth factor receptor tyrosine kinase inhibitor for the targeted therapy of non-small-cell lung cancer (NSCLC) However, the efficacy of erlotinib is limited because the development of drug resistance during chemotherapy. Indoleamine 2,3-dioxygenase-1 (IDO1) is a rate-limiting tryptophan catabolic enzyme that is activated in many human cancers. In this study, we designed a series of erlotinib-based 1,2,3-triazole compounds by combining erlotinib with phenyl or benzyl azide. Attentive FP prediction model was used to predict the bioactivity of those compounds. We discovered that most of the erlotinib-based 1,2,3-triazole compounds are capable of suppressing IDO1 activities *in vitro* experiments. Among them, compound **14b** (IC_50_ = 0.59 ± 0.05 μM) had the strongest inhibitory effect on IDO1. In addition, compound **14b** significantly inhibited tumor growth comparable to the antitumor activity of erlotinib and the IDO1 inhibitor epacadostat in murine tumor models.

## 1 Introduction

**FIGURE 1:**
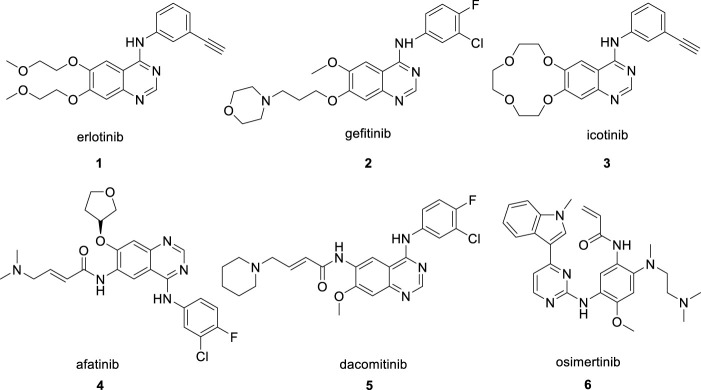
The reported EGFR inhibitors.

**FIGURE 2:**
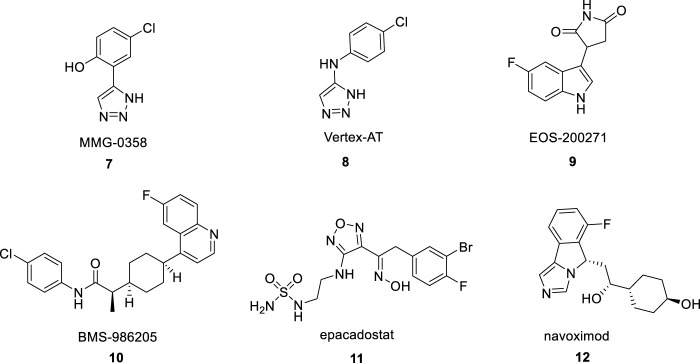
The reported IDO1 inhibitors.

**FIGURE 3:**
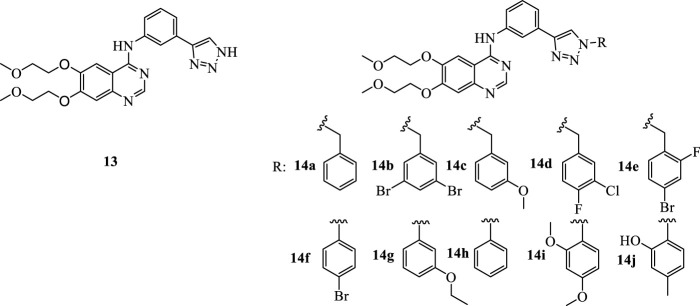
The structures of compound **13** and compounds **14a-14j**.

**FIGURE 4:**
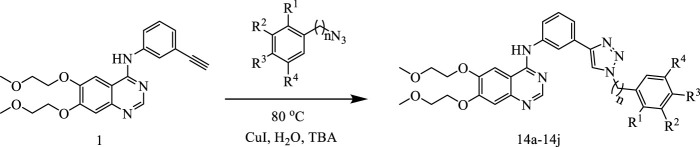
The reaction routes to erlotinib-1,2,3-triazole derivatives.

**FIGURE 5:**
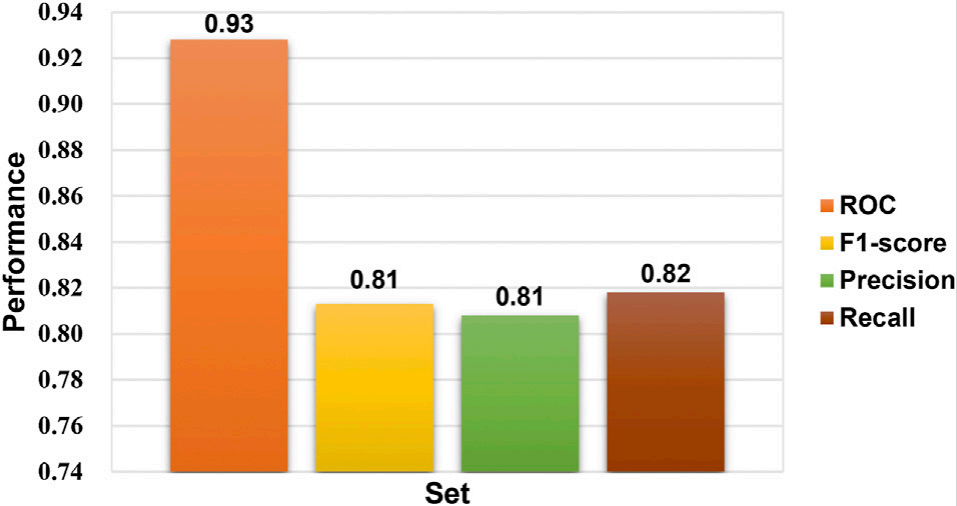
Performance of the Ido1 bioactivity model.

**FIGURE 6:**
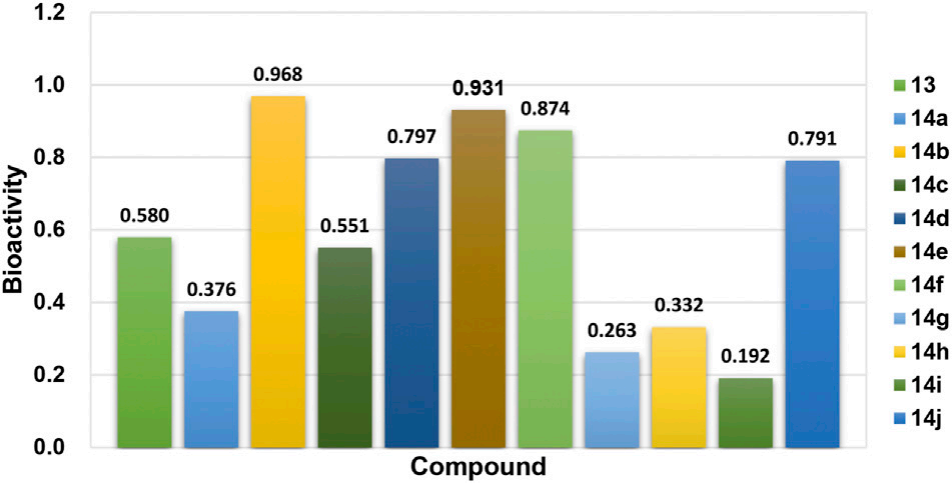
Bioactivity prediction results of the designed compounds.

Erlotinib (Figure 1, **1**) is a standard EGFR inhibitor that was approved for sale by the FDA in 2004(Smith, 2005). As an orally administered small-molecule competitive reversible EGFR-TKI, erlotinib blocks the phosphorylation of EGFR by competing with mutant EGFR for ATP binding and inhibits activation of downstream signaling pathways (Mao et al., 2022). It was initially developed for the first-line treatment of advanced NSCLC caused by EGFR mutations in cases where conventional chemotherapy failed to provide an effective resolution (Hirsch et al., 2017). Compared with clinical treatment by conventional anticancer drugs, erlotinib treatment improves the median overall survival (OS) of patients from 4 months to >40 months and has better performance in the objective response rate (ORR), progression-free survival (PFS) and tolerance (Mathew et al., 2015; Wei et al., 2020). Although erlotinib significantly delays cancer progression in the targeted treatment of NSCLC, like the other two first-generation EGFR inhibitors, gefitinib (Cohen et al., 2003; Zhang et al., 2017), (Figure 1, 2) and icotinib(Shi et al., 2013; Mao et al., 2020) (Figure 1, 3), drug resistance starts to appear after about 9–14 months of application, and almost all tumors begin to re-grow(Ayyappan et al., 2013). The drug resistance problem has persisted with second-generation and third-generation EGFR inhibitors developed subsequently, such as afatinib (Figure 1, 4), dacomitinib (Figure 1, 5) and osimertinib (Figure 1, 6) (Jänne et al., 2015; Qin et al., 2016). Searching for an effective solution to the drug resistance problem of EGFR inhibitors, including erlotinib, remains the major effort of pharmaceutical research (Sun et al., 2022).

**FIGURE 7:**
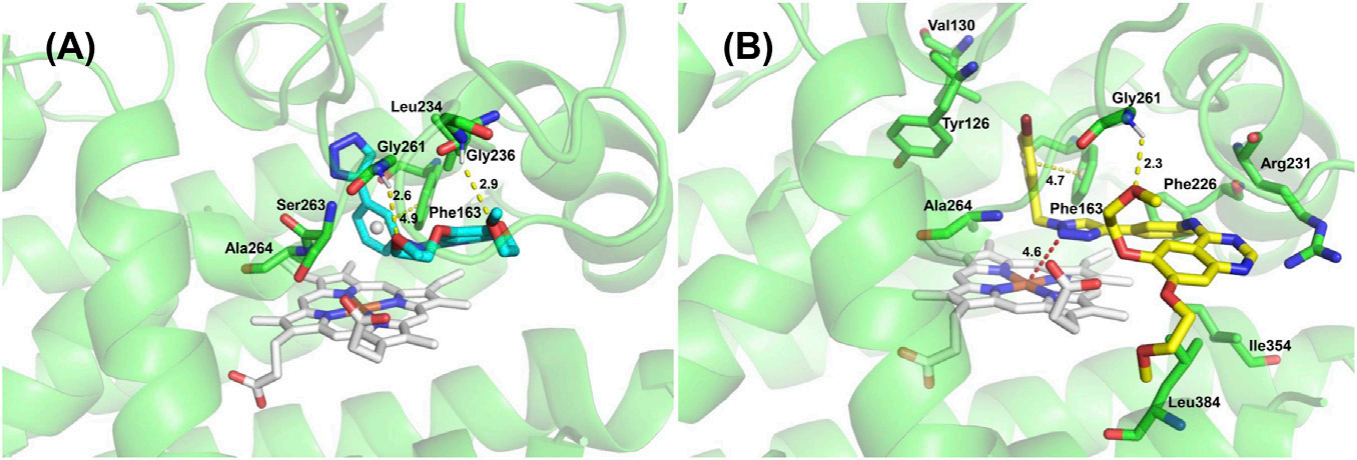
3D binding modes of compound 13 **(A)** and compound 14b **(B)** in complex with IDO1.

**FIGURE 8:**
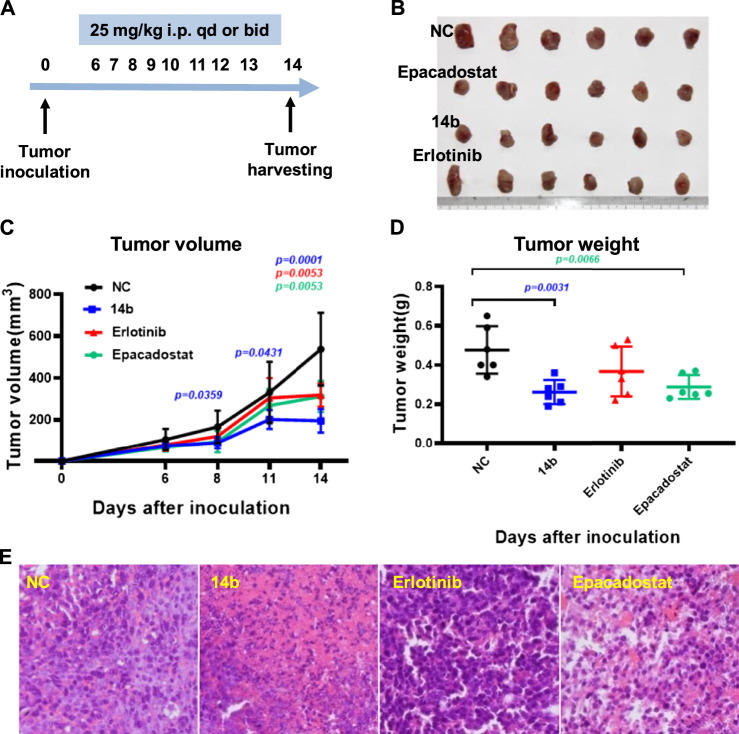
Effects of compound **14b** on 4T1 allograft tumor growth in BALB/c mice. **(A)** Experimental procedure of the murine 4T1 breast model. **(B)** Growth morphology for the 4T1 tumors from the BALB/c mice after treatment with vehicle, compound **14b**, Erlotinib and Epacadostat (*n* = 10, 9, 10 and 10, respectively). **(C)** Tumor volumes formed by 4T1 cells 14 days after inoculation (*n* = 6). **(D)** Tumor weight of 4T1 allograft were presented (*n* = 6). **(E)** Compound **14b**-induced extensive necrosis within tumors detected by Hematoxylin and Eosin (H&E) staining.

**FIGURE 9:**
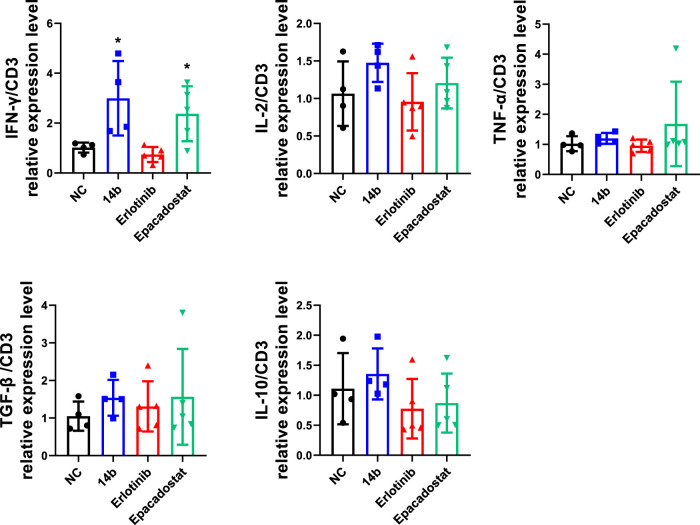
Compound **14b** regulates T cell function-associated genes in TME. Cytokines associated with T cell functions including IFN-γ, IL-2, TNF-α, TGF-β and IL-10 within 4T1 tumors were analyzed by qPCR.

**FIGURE 10:**
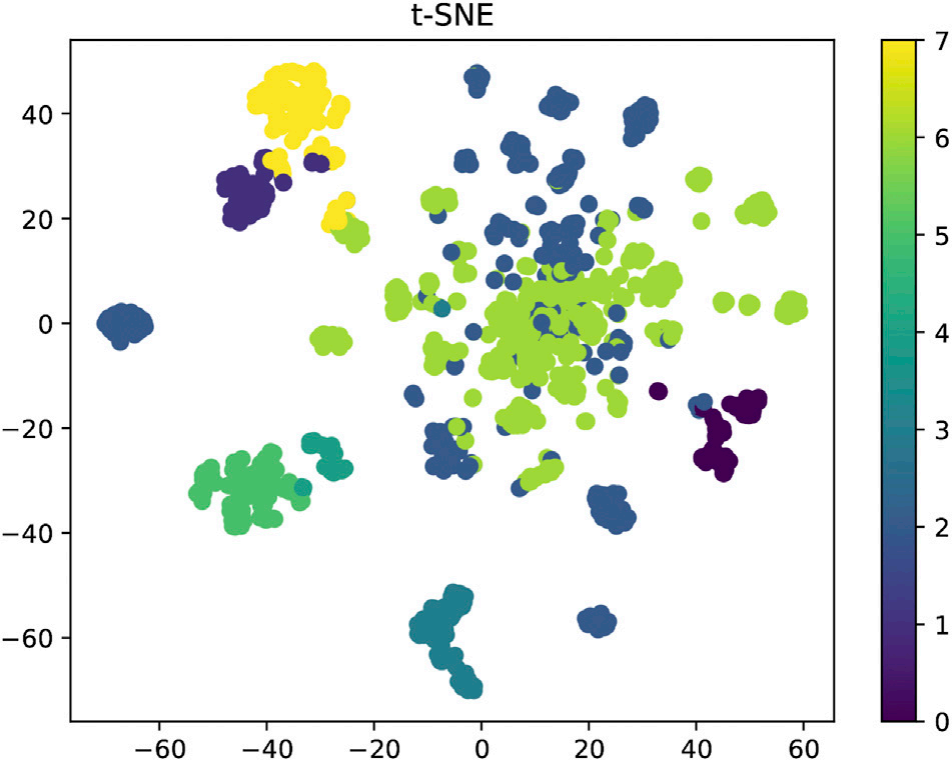
Distribution of IDO1 activity dataset based on pIC_50_.

1,2,3-triazole structural building blocks exhibit a wide range of biological activities in marketed drugs, such as antimicrobial, antitumor (Deng et al., 2022), antitubercular, and antidiabetic effects (Maddili et al., 2018). In addition, abundant studies have shown various 1,2,3-triazole-containing hybrids, such as MMG-0358 (Figure 2, 7) (Röhrig et al., 2010) and Vertex-AT (Figure 2, 8) (Panda et al., 2019) also suppress IDO1 through structure-activity relationship and molecular docking studies. IDO1 is a promising cancer therapeutic target that over expressed in many human cancers (Theate et al., 2015). Especially in lung cancer, IDO1 is a driver of disease progression and metastasis, and is associated with poor prognosis (Smith et al., 2012; Wang et al., 2018). IDO1, which is expressed in multiple types of malignancies from the tumor microenvironment (TME), catalyzes the oxidation of tryptophan (TRP) to immunosupressive metabolite N-formylkynurenine. TRP depletion and catabolite production by IDO1 suppress the function of T effect or cells and natural killer (NK) cell, drive dendritic cells (DCs) (Eric et al., 2018) and macrophages toward an immunosuppressive phenotype, increase the activities of CD4^+^ T regulatory cells (Treg) and myeloid-derived suppressor cells (MDSCs), and sustain an immmnosuppressive environment in TME (Munn and Mellor, 2016; Schupp et al., 2019). Consequently, great efforts have been made to explore IDO1 inhibitors as the promising therapeutic candidate for cancer therapy. The clinical-stage IDO1 inhibitors such as EOS200271 (Figure 2, 9), BMS-986205 (Figure 2, 10), Epacadostat (Figure 2, 11) and Navoximod (Figure 2, 12), bind to IDO1 through different mechanisms (Platten et al., 2019).

The emerging evidence of IDO1 inhibitors in cancer immunotherapy provides ideas for the transformation of erlotinib. On the basis of erlotinib, Professor Yang Qing from Fudan University developed a compound with a triazole structure through click chemistry transformation (Figure 3, **13**). The resulted compound displayed the inhibitory activity on the IDO1 enzyme (IC_50_ = 12.6 μM). Their study expands the application of the erlotinib structure in the development of tumor immunotherapy drugs. In our study, various substituents were introduced on the N atom of the 1-position of triazoles, including five benzyl derivatives substituted compounds (Figure 3, 14a-14e) and five phenyl derivatives substituted compounds (Figure 3, 14f-14j), with the hope to further improve the inhibitory activity against IDO1. In the current era of big data, as a new type of drug research and development technology, deep learning has been involved in multiple stages of drug research. To evaluate the biological activity of our designed molecules, we used the activity prediction function of the molecular representation model Attentive FP (Xiong et al., 2019) to predict the 10 synthesized compounds, so as to evaluate whether the molecules have the biological activity against IDO1. Attentive FP is a graph neural network model based on attention mechanism, which can be used for molecular property prediction, and it performs well in various property prediction results. Compound **13** was used as a control, the promising compounds as supported by Attentive FP were chosen for the further investigation of IDO inhibition, toxicity and tumor inhibitory property using *in vitro* and *in vivo* tumor models.

## 2 Results and discussion

### 2.1 Chemistry

The synthetic route for the preparation of the target compounds was showed in Figure 4. Copper(I)-catalysedazide–alkyne cycloaddition between compound **1** (erlotinib) and different azido compounds produced the target compounds **14a-14j** (Table 1). The structures of all the target compounds were confirmed by nuclear magnetic resonance (^1^H NMR and ^13^C NMR).

**TABLE 1:** The structures of erlotinib-1,2,3-triazole derivatives.

Compd no.	n	R^1^	*R* ^2^	R^3^	R^4^
**14a**	1	H	H	H	H
**14b**	1	H	Br	H	Br
**14c**	1	H	OCH_3_	H	H
**14d**	1	H	Cl	F	H
**14e**	1	F	H	Br	H
**14f**	0	H	H	Br	H
**14g**	0	H	OCH_2_CH_3_	H	H
**14h**	0	H	H	H	H
**14i**	0	OCH_3_	H	OCH_3_	H
**14j**	0	OH	H	CH_3_	H

### 2.2 Bioactivities prediction analysis

In the bioactivity prediction task of Attentive FP, the IDO1 dataset was randomly split according to the ratio of train, valid and test (8:1:1). The evaluation of the model obtained by training were shown in Figure 5. The AUC, F1-score, Precision and Recall are all above 0.8, and the IDO1 dataset showed promising results on Attentive FP. A series of compounds **14a-14j** were designed based on the initial compound **13**, and then the designed compounds were tested using the trained best model. The test results were shown in Figure 6. The probability of predicted bioactivity for compound **13** was at a medium level (0.580). The probability for compounds 14b, 14e and 14f were much higher than compound 13. Among them, compound **14b** had the best bioactivity with a probability of 0.968. Compound 14e and compound 14f were at 0.931 and 0.874. The probability for Compounds **14d** and **14j** were at lower level compared to above group, while higher than compound 13. Compounds **14a**, **14c**, **14g**, **14h** and **14i** were predicted to have weaker activity and activity levels were weaker than the compound **13**. Based on this test result, we predict the designed compounds have bioactivities targeting IDO1, and justified further experimental studies in terms of synthesis and biological activity, especially compound **14b** showed the best activity level.

### 2.3 Ido1 inhibition and cytoxicity assays

To confirm the IDO1 inhibitory activities of the synthesized derivatives, all the compounds were screened using Hela cells as previously described (Malachowski et al., 2016; Qian et al., 2016). Cytotoxicity of compounds was performed by SHEE cell-based MTT assay (Xu et al., 2022). **Epacadostat** was chosen as a positive control with IC_50_ value of 90.7 nM, consistent with previously reported data (IC_50_ = 71.8 nM) (Nelp et al., 2018). The IDO1 inhibitory activities of synthesized compounds were shown in Table 2, indicating that most of the new compounds exhibited moderate to good IDO1 inhibitory activities (IC_50_ values <1.0 μM), particularly compound **14a** (IC_50_ = 0.68 ± 0.42 μM) and compound **14b** (IC_50_ = 0.59 ± 0.05 μM). The inhibition of most compounds on Hela cells exceeded 50 μM except for compound **14e** (IC_50_ = 30.36 ± 3.61 μM). As a conclusion, these compounds could directly inhibit the activities of IDO1 enzyme without damaging Hela cells. Moreover, to test whether the inhibition of IDO1 by the compounds would produce cytotoxicity, we examined the growth inhibition of these compounds against SHEE cells. These results indicated that most compounds exhibited low inhibitory effects on SHEE cells. Therefore, we selected compound **14b** to proceed for anti-tumor experiments *in vivo* considering its good IDO1 inhibitory activity and low cytotoxicity.

**TABLE 2:** IDO1 inhibitory activities of synthesized compounds.

Compd no.	IC_50_ (μM)		
	Ido1 (24 h)	Hela (24 h)	SHEE (48 h)
**14a**	0.68 ± 0.42	>50	>50
**14b**	0.59 ± 0.05	>50	>50
**14c**	2.82 ± 1.87	>50	>50
**14d**	1.24 ± 0.29	>50	47.31 ± 2.97
**14e**	0.97 ± 0.14	30.36 ± 3.61	>50
**14f**	0.92 ± 0.36	>50	7.20 ± 0.63
**14g**	3.12 ± 0.65	>50	22.59 ± 2.31
**14h**	2.61 ± 0.42	>50	>50
**14i**	1.18 ± 0.25	>50	28.08 ± 2.47
**14j**	0.72 ± 0.05	>50	>50
**Epacadostat**	0.0907 ± 0.019	—	—

IC_50_ values were fitted from single point inhibition curves, and two parallel experiments were performed for each compound. IC_50_ values were calculated using Graph Pad Prism 8.0 software. These results are reported as the averages ±SD.

### 2.4 Molecular docking studies of compounds 13 and 14b

Molecular docking is a computational modeling method that can explain the binding mechanism of IDO1 and compounds at the molecular level. Docking result depicted that the binding affinities of compounds **13** and **14b** were both -9.0 kcal/mol. Figure 7 showed that compounds **13** and **14b** were docked into the binding site of IDO1. The amino groups of Gly236 and Gly261 formed hydrogen bond with the oxygen of the ether chain of compound **13**. The amino group of Gly261 formed a hydrogen bond with an oxygen of the ether chain of compound **14b**. Both the quinazoline ring of compound **13** and the 1,3-dibromobenzyl group of compound **14b** could form π-π stacking with Phe163. Additionally, the 1,3-dibromobenzyl of compound **14b** was located in the hydrophobic pocket formed by Tyr126, Val130, Phe163, Ala264 and Gly261. The quinazoline ring of compound **14b** was located in the active pocket formed by Phe226 and Arg231. According to Tojo et al., the interaction of ligand Amg-1 (Co-crystal ligand of 4PK5) with Phe226 and Arg231 is critical for the inhibitory activity of IDO1 (Tojo et al., 2014). The docking results indicated that compound **14b** had better IDO1 inhibitory activity.

### 2.5 *In vivo* antitumor activity

The immunosuppressive effects of IDO1 promote resistance to cancer immune therapy. To better evaluate the role of IDO1 inhibitory activities of **14b** in antitumor effects *in vivo*, we assessed the antitumor efficacy of **14b** on an IDO1-overexpressing murine 4T1 breast model formed in immunocompetent BALB/c mice. The BALB/c mice harboring 4T1 tumors were intraperitoneally injected with **14b** (25 mg/kg, qd), Erlotinib (25 mg/kg, qd) or well-known IDO1 inhibitor Epacadostat (25 mg/kg, bid) for 8 days, and then the tumors were harvested and weighed 14 days after inoculation (Figure 8A). As shown in Figure 8C, 14b and Epacadostat could efficiently suppress the 4T1 tumor growth (Figures 8B,D). Statistical analysis showed that 14b significantly inhibited tumor growth at day 8, 11 and 14 (*p = 0.0359*, *p = 0.0431 and p = 0.0001*) compared to NC group. While the results demonstrated that Erlotinib reduced the weight of the 4T1 tumors at day 14 post inoculation, the tumor volumes were not significantly changed compared to control group. Additionally, necrosis phenomenons such askaryolysis and karyorrhexis were frequently observed in tumor tissues exposed to 14b (Figure 8E).

To further explore the effects of **14b** on antitumor immune response, cytokines associated with T cell functions within the 4T1 tumors were determined by qPCR. Interferon gamma (IFN-γ), Interleukin-2 (IL-2) and tumor necrosis factor (TNF-α) released by Th1 cells are typically associated with cytotoxic function, while transforming growth factor beta (TGF-β) and interleukin-10 (IL-10) driver T cells towards immune suppressive phenotypes in TME. Statistical analysis showed that **14b** and Epacadostat significantly up regulated the RNA levels of IFN-γ within the tumors but the other cytokines were not affected obviously (Figure 9). These data indicated that **14b** has emerged as a potential IDO1 inhibitor comparable to the clinical-stage IDO1 inhibitor. Besides, **14b** increased the expression of IFN-γ in the TME, which was previously reported by other IDO1 inhibitors, and IFN-γ was associated with improved T cell function.

## 3 Conclusion

In conclusion, we designed and synthesized a series of erlotinib-based 1,2,3-triazole derivatives as potential IDO1 inhibitors. Attentive FP prediction results indicated that the inhibition of compound **14b** against IDO1 was better than that of compound **13** (a reported IDO1 inhibitor). All designed compounds were evaluated by measuring the inhibitory activity of IDO1 using Hela cells and SHEE cells. Compound **14b** (IC_50_ = 0.59 ± 0.05 μM) was found to have robust potency and low toxicity for IDO1 inhibition. *In vivo* antitumor studies shown that compound **14b** could significantly inhibit the expression of IDO1 in mice with 4T1 tumor cells, as a result inhibited the tumor growth. Furthermore, the results of immune response experiments indicated that compound **14b** could considerably up-regulate the RNA level of IFN-γ in tumors, thereby improving T cell function. These results warrant compound **14b** for structural optimization and further study as a potential IDO1 inhibitor.

## 4 Experimental protocols

### 4.1 Chemistry

The target compounds were in-house synthesized. All reagents and solvents were purchased from Aladdin’s reagent or Sinopharm Group (China). ^1^H NMR spectra were acquired in DMSO-d_6_ solution with a Bruker600 or Bruker400 spectrometer. Hela cell line, DMEM medium and fetal bovine serum were purchased from ATCC(Virginia, United States).

#### 4.1.1 The preparation of compound 14a-14j

Compound 1 (erlotinib, 1.0 mmol) and trinitrides (1.2 mmol) and cuprousiodide (0.1 mmol) were added to a mixed solvent (V_H2O_:V_(CH3)3COH_ = 2:1, 30 ml).The reaction mixture was stirred for 10 h at 80°C. The mixture was extracted with DCM (dichloromethane, 20 ml × 3). The organic phase was washed successively with brine, then drying with Na_2_SO_4_ and desolventizing. The concentrate was purified by going through column chromatography to obtain the target compounds.

**Compound 14a.** Yield 69%. ^1^H NMR (600 MHz, DMSO-*d*_*6*_): *δ*9.56 (s, 1H), 8.67 (s, 1H), 8.49 (s, 1H), 8.27 (s, 1H), 7.92 (t, J_1_ = 6.0Hz, J_2_ = 12.0Hz, 2H), 7.57–7.56 (m, 1H), 7.46 (t, J_1_ = 6.0Hz, J_2_ = 6.0Hz, 1H),7.42–7.35 (m, 5H), 7.24 (s, 1H), 5.67 (s, 2H), 4.33–4.29 (m, 4H), 3.81–3.79 (m, 2H), 3.77–3.75 (m, 4H), 3.39 (s, 3H), 3.36 (s, 3H). ^13^C NMR (150 MHz, DMSO-*d*_*6*_): 156.8, 154.1, 153.4, 148.6, 147.4, 147.1, 140.5, 136.5, 131.4, 129.5, 129.3, 128.7, 128.4, 122.3, 122.1, 120.8, 119.2, 109.4, 108.7, 103.7, 70.6, 70.5, 68.8, 68.5, 58.9, 58.8, 53.5. – MS (ESI): *m/z* (%) = 527 (100) [M + H]^+^.

**Compound 14b.** Yield 75%. ^1^H NMR (600 MHz, DMSO-*d*_*6*_): *δ* 9.58 (s, 1H), 8.72 (s, 1H), 8.49 (s, 1H), 8.28 (s, 1H), 7.92 (t, J_1_ = 12.0Hz, J_2_ = 6.0Hz, 2H), 7.86 (s, 1H), 7.64 (s, 2H), 7.57 (d, J = 6.0 Hz, 1H), 7.48–7.46 (m, 1H), 7.24 (s, 1H), 5.70 (s, 2H), 4.33–4.29 (m, 4H), 3.81–3.79 (m, 2H),3.77–3.75 (m, 2H), 3.39 (s, 3H), 3.36 (s, 3H). ^13^C NMR (150 MHz, DMSO-*d*_*6*_): 156.8, 154.1, 153.4, 148.6, 147.5, 147.2, 140.8, 140.6, 133.7, 131.2, 130.7, 129.5, 123.2, 122.4, 120.8, 119.3, 109.4, 108.7, 103.7, 70.6, 70.5, 68.8, 68.5, 58.9, 58.8, 52.0. – MS (ESI): *m/z* (%) = 683 (100) [M + H]^+^.

**Compound 14c*.*** Yield 42%. ^1^H NMR (600 MHz, DMSO-*d*_*6*_): *δ* 9.61 (s, 1H), 8.66 (s, 1H), 8.27 (s, 1H), 8.01 (s, 1H), 7.91–7.90 (m, 1H), 7.58–7.56 (m, 1H), 7.47–7.45 (m, 1H), 7.33–7.32 (m, 2H), 6.97–6.92 (m, 3H), 5.63 (s, 2H), 4.33–4.27 (m, 4H), 3.80–3.76 (m, 4H), 3.76 (s, 3H), 3.39 (s, 3H), 3.37 (s, 3H). ^13^C NMR (150 MHz, DMSO-*d*_*6*_): 159.9, 156.8, 154.0, 148.6, 147.1, 140.5, 137.9, 131.4, 130.5, 129.5, 122.4, 122.1, 120.9, 120.5, 119.3, 114.3, 114.0, 108.9, 103.8, 87.8, 70.6, 70.5, 68.9, 68.5, 58.9, 58.8, 55.6, 53.5, 22.6. – MS (ESI): *m/z* (%) = 557 (100) [M + H]^+^.

**Compound 14d.** Yield 87%. ^1^H NMR (400 MHz, DMSO-*d*_*6*_): 9.55 (s, 1H), 8.60 (s, 1H), 8.26 (s, 1H), 7.92 (t, J_1_ = 18.0Hz, J_2_ = 12.0Hz, 2H), 7.57 (t, J_1_ = 6.0Hz, J_2_ = 18.0Hz, 2H), 7.49–7.44 (m, 2H), 7.33–7.28 (m, 2H), 5.75 (s, 2H), 4.33–4.28 (m, 4H), 3.81–3.79 (m, 2H), 3.77–3.75 (m, 2H), 3.39 (s, 3H), 3.37 (s, 3H).^13^C NMR (100Hz, DMSO-*d*_*6*_): 163.5, 161.1, 153.7, 148.7, 146.9, 140.4, 134.3, 134.2, 133.0, 131.3, 130.0, 129.5, 122.4, 122.3, 120.9, 119.3, 117.6, 117.4, 115.5, 115.3, 70.5, 68.8, 68.5, 58.8, 50.7. – MS (ESI): *m/z* (%) = 579 (100) [M + H]^+^.

**Compound 14e.** Yield 62%. ^1^H NMR (400 MHz, DMSO-*d*_*6*_): *δ* 9.58 (s, 1H), 8.63 (s, 1H), 8.26 (s, 2H), 7.91 (s, 1H), 7.66–7.56 (m, 3H), 7.55–7.37 (m, 3H), 5.71 (s, 2H), 4.33–4.29 (m, 4H), 3.79–3.77 (m, 4H), 3.40 (s, 3H), 3.37 (s, 3H). ^13^C NMR (100Hz, DMSO-*d*_*6*_): 161.7, 159.2, 148.7, 147.0, 140.4, 132.8, 131.2, 129.5, 122.9, 122.7, 122.6, 122.4, 122.2, 120.9, 119.7, 119.4, 119.3, 70.5, 68.8, 68.5, 58.8, 47.2. – MS (ESI): *m/z* (%) = 623 (100) [M + H]^+^.

**Compound 14f*.*** Yield 58%. ^1^H NMR (600 MHz, DMSO-*d*_*6*_): *δ* 9.63 (s, 1H), 9.37 (s, 1H), 8.51 (s, 1H), 8.38 (s, 1H), 7.98–7.94 (m, 4H), 7.87–7.85 (m, 2H), 7.67–7.65 (m, 1H), 7.54–7.52 (m, 1H), 7.25 (s, 1H), 4.34–4.32 (m, 2H),4.31–4.30 (m, 2H), 3.82–3.80 (m, 2H), 3.77–3.76 (m, 2H),3.39 (s, 3H), 3.37 (s, 3H). ^13^C NMR (150 MHz, DMSO-*d*_*6*_): 156.9, 154.1, 153.4, 148.6, 148.0, 147.4, 140.6, 136.3, 133.3, 130.8, 129.6, 122.8, 122.4, 121.8, 121.0, 120.2, 119.5, 109.4, 108.7, 103.7, 70.6, 70.5, 68.8, 68.5, 58.9, 58.8. – MS (ESI): *m/z* (%) = 591 (100) [M + H]^+^.

**Compound 14g*.*** Yield 88%. ^1^H NMR (600 MHz, DMSO-*d*_*6*_): *δ* 9.63 (s, 1H), 9.36 (s, 1H), 8.51 (s, 1H), 8.37 (s, 1H), 7.96–7.95 (m, 2H), 7.67–7.66 (m, 1H), 7.58–7.56 (m, 1H), 7.55–7.52 (m, 3H), 7.25 (s, 1H), 7.08–7.07 (m, 1H), 4.34–4.32 (m, 2H),4.31–4.30 (m, 2H), 4.16 (dd, J_1_ = 6.0Hz, J_2_ = 6.0Hz,2H), 3.82–3.80 (m, 2H), 3.77–3.76 (m, 2H), 3.39 (s, 3H), 3.37 (s, 3H), 1.40–1.38 (m, 3H). ^13^C NMR (150 MHz, DMSO-*d*_*6*_): 159.9, 156.9, 154.1, 153.4, 148.6, 147.8, 147.5, 140.6, 138.1, 131.4, 131.0, 129.6, 122.8, 121.0, 120.1, 119.5, 115.3, 112.3, 109.5, 108.7, 106.5, 103.7, 70.6, 70.5, 68.8, 68.5, 64.1, 58.9, 58.8, 15.0. – MS (ESI): *m/z* (%) = 557 (100) [M + H]^+^.

**Compound 14h*.*** Yield 71%. ^1^H NMR (600 MHz, DMSO-*d*_*6*_): *δ* 9.63 (s, 1H), 9.34 (s, 1H), 8.50 (s, 1H), 8.38 (s, 1H), 8.00–7.94 (m, 4H), 7.68–7.64 (m, 3H), 7.54–7.51 (m, 2H), 7.24 (s, 1H), 4.34–4.32 (m, 2H),4.31–4.29 (m, 2H), 3.81–3.80 (m, 2H), 3.77–3.76 (m, 2H), 3.39 (s, 3H), 3.36 (s, 3H). ^13^C NMR (150 MHz, DMSO-*d*_*6*_): 156.9, 154.1, 153.4, 148.6, 147.8, 147.5, 140.6, 137.1, 131.0, 130.4, 129.6, 129.2, 122.7, 121.0, 120.5, 120.2, 119.5, 109.4, 108.7, 103.7, 70.6, 70.5, 68.8, 68.5, 58.9, 58.8.—MS (ESI): *m/z* (%) = 513 (100) [M + H]^+^.

**Compound 14i.** Yield 48%. ^1^H NMR (400 MHz, DMSO-*d*_*6*_): *δ* 9.66 (s, 1H), 8.88 (s, 1H), 8.55 (s, 1H), 8.39 (s, 1H), 8.02–7.98 (m, 1H), 7.97–7.92 (m, 1H), 7.71–7.70 (m, 1H), 7.63–7.62 (m, 1H), 7.55–7.73 (m, 1H), 7.29 (s, 1H), 6.92–6.91 (m, 1H), 6.79–6.75 (m, 1H), 4.39–4.37 (m, 2H), 4.36–4.34 (m, 2H), 3.93 (s, 3H), 3.92 (s, 3H), 3.87–3.80 (m, 4H), 3.44 (s, 3H), 3.42 (s, 3H). ^13^C NMR (100 MHz, DMSO-*d*_*6*_): 161.7, 156.8, 154.0, 153.7, 153.4, 148.5, 146.5, 140.5, 131.3, 129.5, 127.5, 124.1, 122.5, 121.0, 119.6, 119.3, 108.6, 105.7, 103.6, 99.9, 70.6, 70.5, 68.8, 68.5, 58.9, 58.8, 56.7, 56.2.—MS (ESI): *m/z* (%) = 573 (100) [M + H]^+^.

**Compound 14j*.*** Yield 71%. ^1^H NMR (400 MHz, DMSO-*d*_*6*_): *δ* 9.66 (s, 1H), 8.98 (s, 1H), 8.55 (s, 1H), 8.40 (s, 1H), 8.00–7.98 (m, 2H), 7.75–7.71 (m, 2H), 7.63 (t, J = 6.0 Hz, 1H), 7.55 (t, J = 6.0 Hz, 1H), 7.41–7.40 (m, 1H), 7.29 (s, 1H), 7.24 (t, J = 6.0 Hz, 1H), 4.39–4.37 (m, 2H), 4.36–4.34 (m, 2H), 3.95 (s, 3H), 3.87–3.80 (m, 4H), 3.44 (s, 3H), 3.42 (s, 3H). ^13^C NMR (100 MHz, DMSO-*d*_*6*_): 156.9, 154.0, 153.4, 152.3, 148.5, 147.4, 146.7, 140.5, 131.4, 131.2, 129.5, 126.5, 126.2, 124.0, 122.6, 121.4, 121.0, 119.4, 113.5, 109.4, 108.6, 103.6, 70.6, 70.5, 68.9, 68.5, 58.9, 58.8, 56.6.—MS (ESI): *m/z* (%) = 543 (100) [M + H]^+^.

### 4.2 Bioactivities prediction based on attentiveFP

We used Attentive FP to predict bioactivities for the designed compounds. Firstly, we obtained the activity dataset of IDO1 from the ChEMBL database (1785 molecules), which used pIC_50_ (negative logarithm of IC_50_) as an indicator of activity. IDO1 activity dataset was split into positive and negative sets by setting a threshold of 6.0. Before training the model, we used k-means clustering and t-SNE dimensionality reduction to display the distribution of IDO1 activity data, as shown in Figure 10. In this work, IDO1 dataset was used to train the activity prediction classification model. The model used the Adam optimizer for gradient descent to maintain the best parameters of the model. After training for 800 epochs, the best model for the IDO1 target was output, and finally this model was used to test the activity probability of our designed compounds against IDO1. This model used CrossEntropyLoss to measure the cross entropy to calculate the loss function. The AUC, F1-score, Precision, Recall as performance evaluation indicators.

### 4.3 *In vitro* Ido1 enzymatic inhibition assay

To demonstrate the inhibitory effect of the designed compounds against IDO1, Hela cells were seeded at 50,000∼60,000 cells per well into 96-well plate in 100 μl of dulbecco’s modified eagle medium complete growth medium for 12∼18 h. The second day, 100 μl per well of diluted inhibitor was added at a final concentration of 100 ng/ml human IFNγ, and then incubated at 37°C, 5% CO_2_ for 18 h. The third day, 140 μl of medium was removed into a new 96-well plate and precipitate the protein with 10 μl of 6.1 N TCA (CCl_3_COOH) at 50°C for 30 min. The plate was centrifuged at 2,500 rpm for 10 min. Then 100 μl of supernatant per well was transferred to another 96-well plate and mixed with 100 μl of p-Dimethylaminobenzaldehyde in CH_3_COOH [2% (w/v)]. The plate was incubated at 25°C for 10 min, the yellow color derived from kynurenine was recorded by measuring absorbance at 480 nm using a microplate reader (PE, United States).

### 4.4 Cytoxicity assay

We used the Cell Counting Kit-8 (CCK-8, Meilunbio, Dalian, China) assay to evaluate whether compounds directly inhibit IDO1 in Hela cells. Make 1,000× compounds solution in DMSO. Add 1 μl 1,000× compounds to 49 μl growth medium to make 20× compounds. Dilute cell suspensions in growth medium to desired density and 95 μl were taken to 96-well plate. Add 5 µl 20× compounds into 96-well plate according to the plate map. Final DMSO concentration in each well was 0.1%. Then the cell was incubated at 37°C, 5% CO_2_ for 24 h. The next day, 20 μl CCK-8 solution was added into each well. After 2 h of incubation at 37°C, the plate was measured at 450 nm by an EnVision Multilabel Reader (PerkinElmer).

Cytoxicity of compounds against SHEE cells was evaluated by MTT assay. The SHEE cells were seeded in 96-well plates with densities of 2,200 − 2,500 cells/well in 100 μl. One day after seeding, the concentration of the test compounds being between 0 and 50 μM, 0.1% DMSO were added to the cells as control. Approximately 2,200 − 2,500 transfected cells in 100 μl were incubated in quintuplicate in 96-well plates. After 48 h, MTT was added and incubated in the plate for 1–4 h in the incubator. The absorbance at 490 nm was measured using a microplate reader (Thermo).

### 4.5 Molecular docking

In this study, we used AutoDockTools (ADT) 1.5.6 software to process protein and ligands, and then constructed the parameter files required for docking (Morris et al., 2009). The crystal structure of IDO1 was obtained from the RCSB PDB database for docking modeling (PDB code:4PK5). IDO1 Protein was prepared by removing water, adding polar hydrogens, calculating Gasteiger charges and assigning AD4 type. Ligands were constructed by ChemDraw and optimized by MMFF94 and MM2 force fields, and then the torsional degrees of freedom of the ligands were detected by ADT. AutoDock Vina was used to perform molecular docking operations (Trott and Olson, 2010). Protein-Ligand Interaction Profiler (PLIP) web tool and PyMol software were used to visualize the interactions of IDO1 and designed compounds (Adasme et al., 2021).

### 4.6 *In vivo* antitumor assay of compound 14b

4T1 allograft were constructed by subcutaneous transplantation of cancer cells into the flanks of 4–6 weeks old BALB/c mice. Tumor volumes were measured using calipers and calculated as above, all mice were assigned randomly to four groups: one group was intraperitoneally injected with vehicle (5% DMSO, 30% PEG300 and 65% ddH_2_O; qd), one group was injected with compound **14b** (25 mg/kg, qd), one group was injected with Erlotinib (25 mg/kg, qd) and one group received daily injections of Epacadostat (25 mg/kg, bid) in vehicle for eight consecutive days. Two weeks after inoculation, the mice were executed and the tumor tissues were harvested and weighed. Hematoxylin and Eosin (H&E) staining for the tumors was performed as we previously described (Xu et al., 2016). A piece of tumor was randomly selected for RNA extraction and RNA was converted to cDNA according to the manufacturer’s instructions. The primers for the target genes were shown in Table3 qPCR was performed to quantification of genes including IFN-γ, IL-2, TNF-α, TGF-β and IL-10. Statistical significance was evaluated by the Student’s t-test.

**TABLE 3:** Primer sequences of the target genes.

Gene	Sequence
IFN-γ	F:AGACAATCAGGCCATCAGCA
	R:TGGACCTGTGGGTTGTTGAC
IL-2	F:ATGAACTTGGACCTCTGCGG
	R:GTCCACCACAGTTGCTGACT
TNF-α	F:GATCGGTCCCCAAAGGGATG
	R:TTGCTACGACGTGGGCTACA
TGF-β	F:AGGGCTACCATGCCAACTTC
	R:CCACGTAGTAGACGATGGGC
IL-10	F:AGGCGCTGTCATCGATTTCT
	R:ATGGCCTTGTAGACACCTTGG

4T1 tumors cells were incubated in 12-well plates for 24 h, and then treated with 0.1% DMSO, various concentrations of either compound **14b** for 16 h. Total RNA was extracted from 4T1 tumors cells tissue and cDNA was synthesized according to the manufacturer’s instructions. Quantitative analysis of IFN-γ, IL-2, TNF-α, TGF-β and IL-10 genes by qPCR, the primers of the genes were shown in Table 3 (Mao et al., 2020)*.*

## Data Availability

The original contributions presented in the study are included in the article/Supplementary Materials, further inquiries can be directed to the corresponding authors.
